# Burosumab as bridging therapy in autosomal dominant hypophosphatemic rickets with femoral fracture nonunion and iron deficiency

**DOI:** 10.1093/jbmrpl/ziag151

**Published:** 2026-09-15

**Authors:** Matej Rakusa, Tomaz Kocjan

**Affiliations:** Department of Endocrinology, Diabetes and Metabolic Diseases, University Medical Centre Ljubljana, 1525 Ljubljana, Slovenia; Faculty of Medicine, University of Ljubljana, 1000 Ljubljana, Slovenia; Faculty of Medicine, University of Ljubljana, 1000 Ljubljana, Slovenia; Department of Endocrinology, Diabetes and Metabolic Diseases, University Medical Centre Ljubljana, 1525 Ljubljana, Slovenia

**Keywords:** burosumab, autosomal dominant hypophosphatemic rickets, nonunion fracture, hypophosphatemia, iron deficiency

## Abstract

Autosomal dominant hypophosphatemic rickets (ADHR) is a rare genetic disorder, characterized by fibroblast growth factor 23 (FGF23)-mediated renal phosphate wasting and impaired bone mineralization. Iron deficiency in ADHR increases FGF23 production and can worsen hypophosphatemia. Evidence to guide optimal management in severe adult cases remains limited. We report the use of burosumab as bridging therapy in a 19-yr-old woman with genetically confirmed ADHR (FGF23 p.Arg176Trp), severe osteomalacia, iron-deficiency anemia, and a low-energy femoral diaphyseal fracture complicated by nonunion despite conventional therapy. Treatment with burosumab resulted in rapid clinical improvement, with marked reduction in musculoskeletal pain and stiffness, and restoration of independent ambulation within four months. BMD increased substantially (+11.7% at the LS and +14.2% at the total hip at 5 mo; +63.8% and +55.5% from baseline at approximately 11 mo, respectively), while trabecular bone score remained within the normal range, a pattern consistent with improved mineralization and preserved trabecular texture. Serum phosphate increased but did not consistently normalize at trough, prompting dose escalation. At 8 mo, the burosumab dose was increased to 50 mg (1.25 mg/kg) due to persistent hypophosphatemia; with concurrent oral iron replacement, transient hyperphosphatemia (1.61 mmol/L) occurred and was managed by delaying the next dose by 14 d and reducing the dose back to 1 mg/kg. The patient also underwent additional surgical intervention, which may have contributed to the overall outcome; radiographic callus formation was evident 5 mo post-surgery. To our knowledge, this is the first full-length report describing burosumab use in genetically confirmed ADHR with longitudinal biochemical and densitometric follow-up, and it highlights the importance of careful monitoring of phosphate and iron indices when combining therapies.

## Graphical Abstract

**Graphical Abstract:**
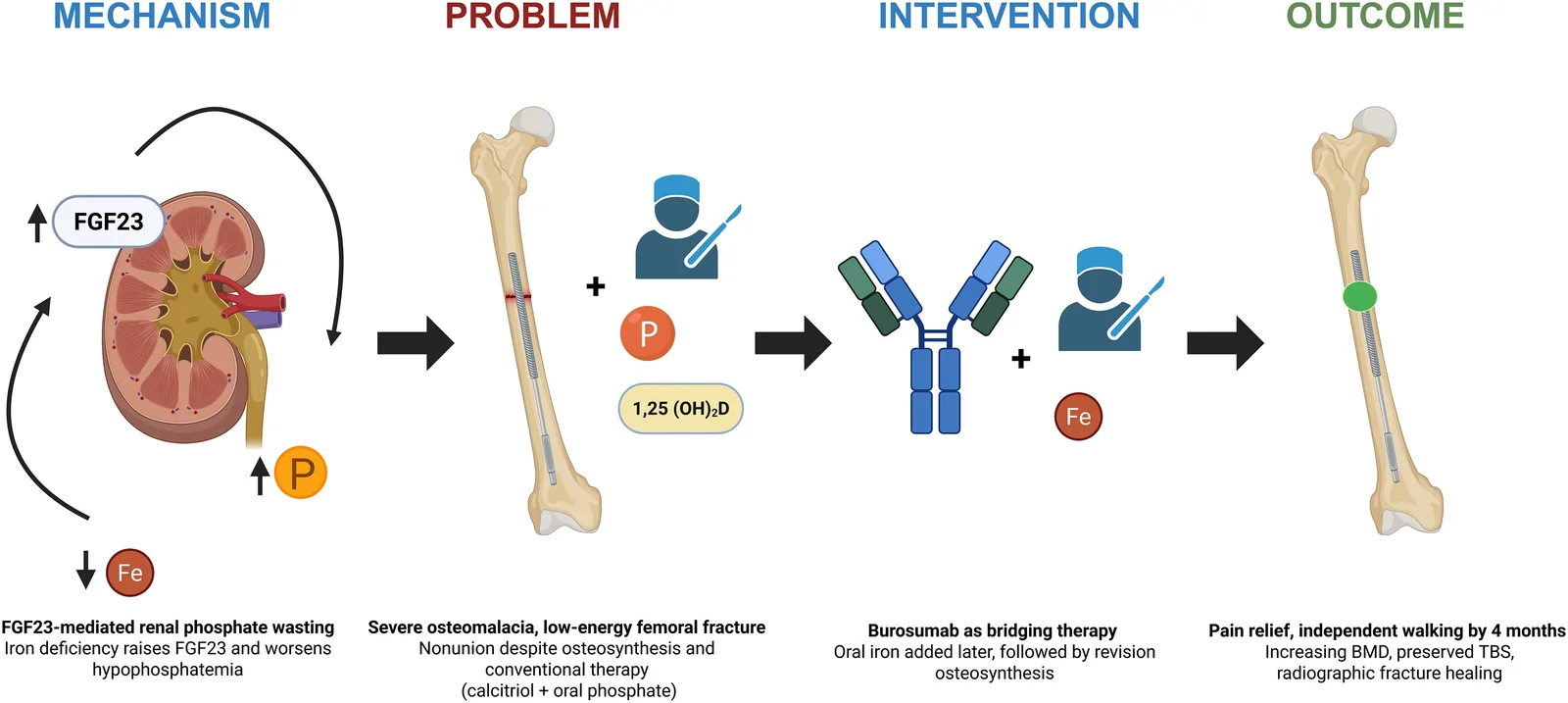

Created in BioRender. Rakusa, M. (2026) https://BioRender.com/0ewh2f2

## Introduction

Hypophosphatemic rickets is a group of rare genetically determined disorders, with fibroblast growth factor 23 (FGF23) mediated, excessive urinary phosphate loss, and impaired bone mineralization. One of the least commonly recognized forms is autosomal dominant hypophosphatemic rickets (ADHR)^1^ that is caused by the mutation disrupting the R176XXR179 protease recognition site in FGF23, and the resultant mutant FGF23 is elevated due to reduced FGF23 cleavage.^2^ The ADHR phenotype, including age of presentation, severity of clinical features, and the potential for spontaneous resolution, is variable and incompletely penetrant. The mutation site may contribute to this variability, as R179 mutations have been associated with an earlier onset than R176 mutations.^3^ Our patient carries the R176W mutation. However, there is also an interaction with an acquired condition, namely iron deficiency, which is unique among the inherited phosphate disorders. Therefore, delayed presentation and spontaneous resolution of the disease have been described, apparently due to differences in iron stores.^2^ FGF23 reduces phosphate reabsorption in the kidneys by reducing sodium-dependent phosphate transport proteins (NaPi-IIa/NaPi-IIc) in proximal tubule brush-border membrane. Furthermore, FGF23 suppresses renal 1α-hydroxylase (CYP27B1) and stimulates 24-hydroxylase (CYP24A1), thereby reducing circulating 1,25(OH)_2_D_3_ concentrations and intestinal phosphate absorption.^4^ As in X-linked hypophosphatemia (XLH), conventional treatment with oral phosphate and active vitamin D analogs can normalize serum phosphate levels, and improve symptoms, bone histomorphometry, and radiographic healing in patients with ADHR. Despite many benefits, disadvantages, such as multiple daily dosing of phosphate, the narrow therapeutic window for active vitamin D analogs, frequent gastrointestinal side effects, possible iatrogenic hyperparathyroidism, and nephrocalcinosis, make treatment challenging.^5^ Iron deficiency upregulates FGF23 transcription in ADHR. Because of resistance to degradation, levels of FGF23 increase. There are reports of successful treatment of iron-deficient ADHR patients with iron replacement leading to normalization of phosphate levels and symptom improvement.^6–8^ However, iron toxicity can develop.^6^ Also, to our knowledge, the effect of iron replacement on fracture healing and BMD in ADHR has not been systematically studied.

Burosumab is a monoclonal blocking antibody that targets FGF23. It has been approved for the treatment of XLH by multiple regulatory agencies, including the U.S. Food and Drug Administration, the European Medicines Agency, Health Canada, Japan’s Ministry of Health, Labour and Welfare, and Australia’s Therapeutic Goods Administration, among others. It is administered by s.c. injection every 4 wk in adults.^9^ In addition to XLH, burosumab is approved for FGF23-mediated hypophosphatemia in tumor-induced osteomalacia, when the tumor cannot be localized or completely resected, by the U.S. FDA, the EMA, and the authorities of Japan, Korea, and China, supported by clinical trial evidence including the study by Jan de Beur et al.^10^

Here, we report the successful use of burosumab as bridging therapy in a 19-yr-old woman with genetically confirmed ADHR and a low-energy femoral fracture, after an inadequate response to conventional treatment.

## Case presentation

This 19-yr-old woman with ADHR transitioned to our outpatient endocrinology clinic, from the pediatric endocrine care, for continued follow-up and treatment. She was diagnosed with ADHR at 14 yr of age. In brief, she was born in the 40th week of an unremarkable pregnancy. Her birth weight was 3450 g, birth length 52 cm, head circumference 34 cm, and Apgar scores were 10/10/10. Progressive growth retardation was noted from 6 yr of age (height percentile declined over time: 6 yr >25th, 7 yr <25th, 8.5 yr <7th, and 11 yr <3rd percentile). Growth hormone deficiency was excluded. An X-ray of the left wrist revealed delayed bone age compared with chronological age (−2.32 SD) with metaphyseal widening and fraying of the radius and ulna. X-rays of both knees showed marked bone demineralization and wide, cupped, and frayed metaphyses. Subsequently, genetic analysis confirmed a mutation in the *FGF23* gene, R176W (NP_065689.1:p.Arg176Trp). Treatment with calcitriol 0.5 μg twice daily and oral phosphate supplementation was initiated, one capsule contained potassium dihydrogen phosphate 625 mg and disodium hydrogen phosphate dihydrate 375 mg, providing approximately 6.7 mmol (≈208 mg) of elemental phosphorus, given twice daily and gradually increased to four times daily. She suffered recurrent patellar dislocation and degeneration of the lateral meniscus without rupture. At the age of 17 yr, she suffered a bilateral fracture of the proximal fibula. At the age of 18 yr, she sustained a low-energy fracture of the right femur diaphysis (December 2021), which was treated with osteosynthesis. The treatment course was complicated by fracture nonunion, recognized in March 2023, and a contracture of the right knee. Additional surgical intervention was planned. Conventional therapy was continued throughout this period, during which she sustained the femoral fracture and developed nonunion, indicating an inadequate response to treatment. The treatment timeline is shown in Figure 1.

**Figure 1:**
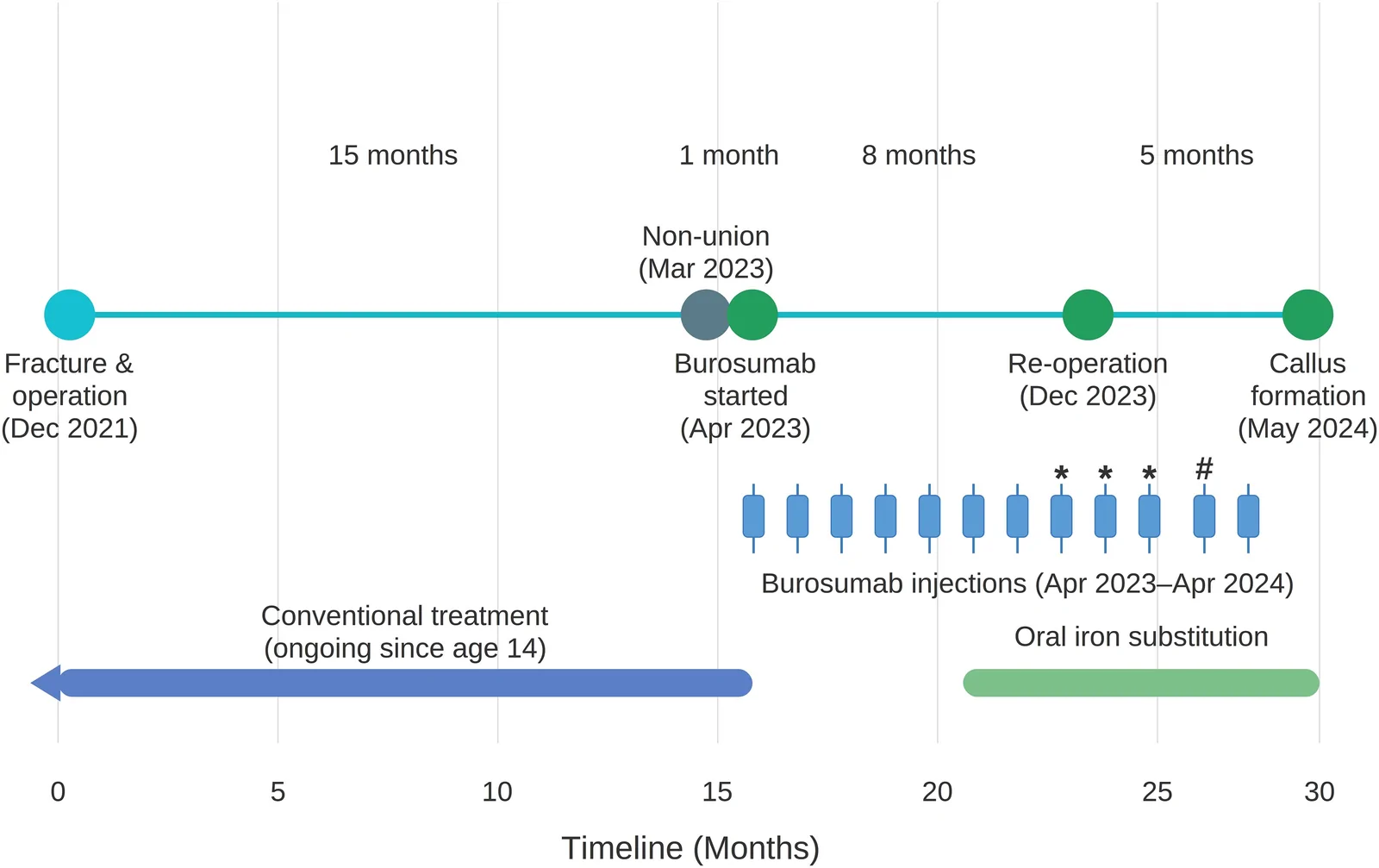
Clinical timeline of a right femoral diaphyseal fracture complicated by nonunion and subsequent management. Month 0 corresponds to the initial fracture treated with osteosynthesis (December 2021). Atrophic nonunion was recognized in March 2023, burosumab was initiated in April 2023, and revision osteosynthesis for persistent nonunion was performed in December 2023. Radiographs demonstrated bridging callus formation in May 2024, while the patient was receiving burosumab. Intervals between events are shown to scale (15, 1, 8, and 5 mo). Horizontal bars indicate the periods of conventional therapy and oral iron supplementation. Each burosumab dose is marked with a syringe icon. Asterisks (*) indicate doses administered at the escalated dose of 1.25 mg/kg body weight. The hash symbol (#) indicates a single dose that was delayed by 14 d (February 2024) because of transient hyperphosphatemia; treatment then continued as scheduled.

On presentation, she relied on crutches for walking due to chronic, moderate, and disabling musculoskeletal pain, rigidity, stiffness, and muscle weakness. At baseline, the patient’s weight was 40 kg, height 145 cm, sitting height 77 cm, and arm span 142 cm, resulting in a BMI of 19.0 kg/m^2^. She had valgus deformity and flexion contracture at the knees, with an interankle distance of 7 cm. The patient reported regular menstrual cycles. Baseline BMD, as assessed by DXA, was below the expected range for age and sex; the trabecular bone score (TBS) was normal. Laboratory tests were notable for low hemoglobin (96 g/L; reference range: 120-150 g/L), serum iron (4.2 μmol/L; 9.0-30.4 μmol/L), and 25OHD (37.7 nmol/L; >75 nmol/L). Serum phosphate (0.39 mmol/L; 0.74-1.52 mmol/L) remained low after long-standing conventional therapy. Alkaline phosphatase (ALP) (4.95 μkat/L; 0.55-1.64 μkat/L) and bone-specific ALP (BALP) (93.6 μg/L; 4.7-27.0 μg/L) were elevated. Procollagen type I N-propeptide (P1NP) was high-normal (114.8 μg/L; 27.7-127.6 μg/L), while CTX (0.334 μg/L; 0.121-0.747 μg/L), corrected total calcium (2.18 mmol/L; 2.10-2.60 mmol/L), and intact PTH (iPTH) (31 ng/L; 18-80 ng/L) were normal. Ferritin was not available at baseline; iron deficiency was suggested by low serum iron and microcytic anemia.

Because of severe, symptomatic disease and a femoral nonunion that had persisted for 15 mo despite conventional therapy, treatment with burosumab (Crysvita) 40 mg (1 mg/kg) s.c. every 4 wk was started in April 2023, after calcitriol and phosphate supplementation had been discontinued 7 d prior. Although burosumab is not approved for ADHR, its use was supported by the common FGF23-mediated mechanism of phosphate wasting. It was therefore prescribed off-label, with reimbursement obtained through an individual request to the national health insurance agency, and the patient provided written informed consent for off-label treatment.

The patient reported a marked reduction in musculoskeletal pain, as well as in stiffness, after 2 mo of treatment. Muscle strength progressively improved, and she was able to walk without crutches by the fourth month. BMD increased markedly after 5 mo of burosumab treatment by 11.7% at the LS and 14.2% at the TH. However, TBS did not change substantially during this period. On the standard dose, serum phosphate peaked at 0.6 mmol/L at 3 mo but did not return to normal, while trough values measured before the next dose were lower (Table 1). The tubular maximum reabsorption of phosphate corrected for glomerular filtration rate (TmP/GFR) and tubular reabsorption of phosphate (TRP) also remained low. TmP/GFR and TRP were calculated from simultaneously obtained fasting serum and fasting second-void urine samples at each assessment. Bone turnover markers, including P1NP, ALP, and BALP, increased, while the CTX increased briefly and then normalized. Laboratory and densitometric parameters during treatment are detailed in Table 1. Revision osteosynthesis for persistent nonunion was performed in December 2023, with radiographic callus formation evident approximately 5 mo later (Figure 2).

**Table 1:** Laboratory parameters and DXA measurements during burosumab therapy.

Burosumab	1. inj./baseline	6. inj./1 mg/kg	10. inj./1.25 mg/kg + iron	Dose delayed (14 d) ^a^	12. inj./follow-up	Reference range
**Date**	19.4.2023	6.9.2023	10.1.2024	February 2024	10.4.2024	
**P (mmol/L)**	0.39	0.36	1.38	1.61	0.62	0.74-1.52
**Corrected total Ca (mmol/L)**	2.18	2.14	2.29	2.35	2.24	2.10-2.60
**ALP (μkat/L)**	4.95	10.31	5.27	-	3.89	0.55-1.64
**BALP (μg/L)**	93.6	284.2	107.5	-	62.4	4.7-27.0
**P1NP (μg/L)**	114.8	>230.0	>230.0	-	>230.0	27.7-127.6
**CTX (μg/L)**	0.334	0.548	0.857	-	0.357	0.121-0.747
**Intact PTH (ng/L)**	31.0	31.0	25.0	-	44	18-80
**25(OH)D (nmol/L)**	37.7	78.4	38.2	-	68.4	>75.0
**1,25(OH)**_**2**_ **D (pmol/L)**	-	28	337	-	94	40-155
**TmP/GFR (mmol/L)**	-	0.26	1.53	-	0.46	0.70-1.35
**TRP (%)**	-	72	93	-	75	85-95
**Creatinine (μmol/L)**	39	32	35	52	39	49-90
**Albumin (g/L)**	51	50	52	-	51	32-55
**Hemoglobin (g/L)**	96	87	145	-	115	120-150
**RBC count (10** ^**12**^ **/L)**	4.63	4.47	5.40	-	4.58	4.50-5.50
**Hematocrit (%)**	33.2	30.1	46.9	-	36.6	36-46
**MCV (fl)**	71.7	67.3	86.9	-	79.9	83.0-101.0
**Iron (μmol/L)**	4.2	2.4	9.3	-	4.2	9.0-30.4
**Ferritin (μg/L)**	-	-	-	-	5	5-204
**BMD L1-L4 (g/cm** ^**2**^**)**	0.544	0.607	-	-	0.891^b^	-
**BMD FN (g/cm** ^**2**^**)**	0.356	0.422	-	-	0.635^b^	-
**BMD TH (g/cm** ^**2**^**)**	0.363	0.414	-	-	0.565^b^	-
**Z-score L1-L4 (SD)**	−4.4	−3.8	-	-	−1.2^b^	>−2.0
**Z-score FN (SD)**	−4.4	−3.8	-	-	−1.9^b^	>−2.0
**Z-score TH (SD)**	−4.7	−4.3	-	-	−3.1^b^	>−2.0
**TBS**	1.358	1.382	-	-	1.420^b^	>1.310

Values represent trough measurements obtained immediately before the next scheduled injection.

^a^The 14-d delay of the next burosumab dose (February 2024) was prompted by transient hyperphosphatemia following the dose increase and concurrent oral iron; treatment then continued as scheduled.

^b^The follow-up DXA was done after approximately 11 mo (March 6, 2024).

Abbreviations: ALP, alkaline phosphatase; BALP, bone specific alkaline phosphatase; Ca, calcium; P, phosphate; TBS, trabecular bone score; TmP/GFR, tubular maximum reabsorption of phosphate corrected for glomerular filtration rate of fasting second-void urine; TRP, tubular reabsorption of phosphate.

**Figure 2:**
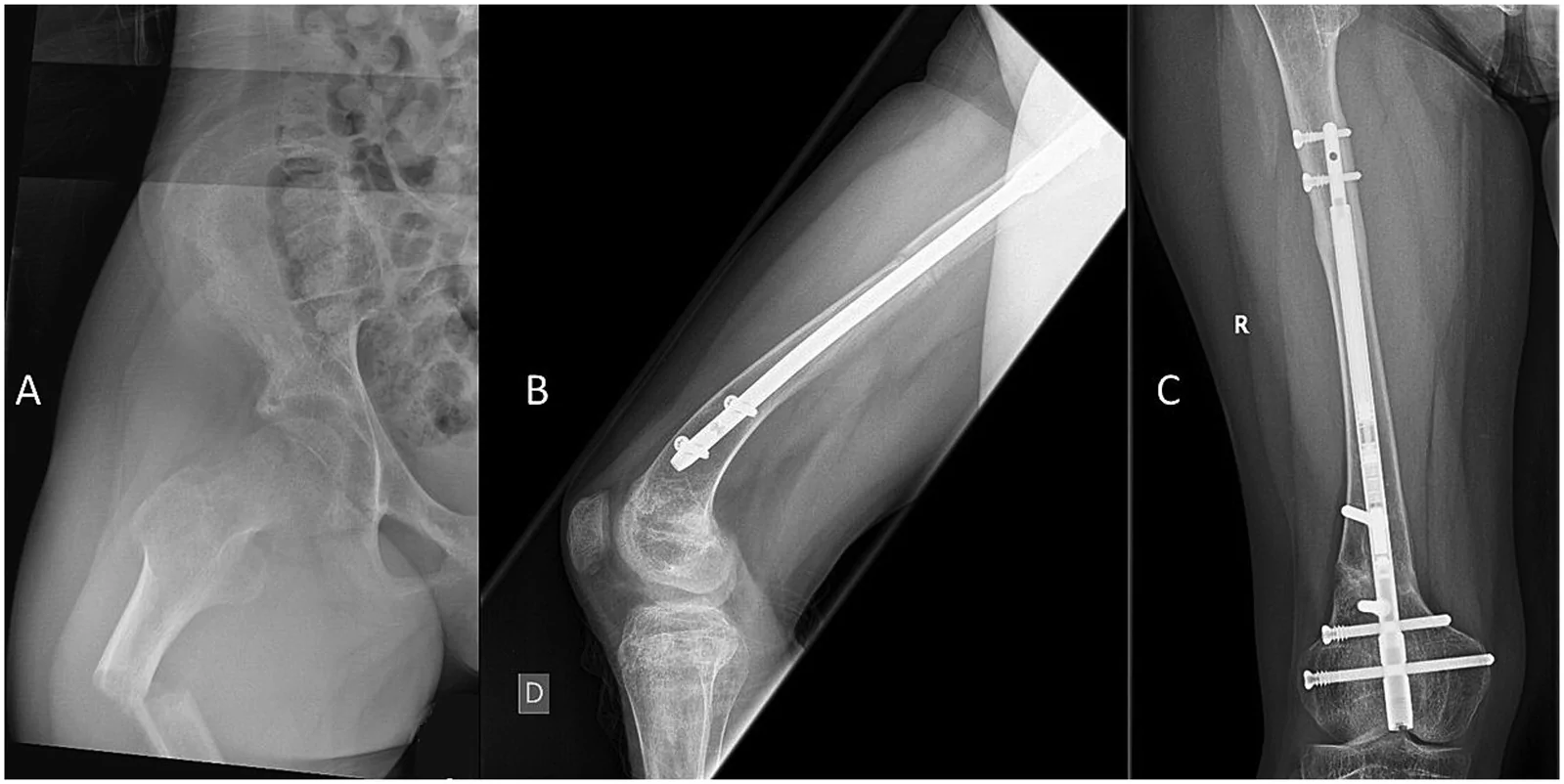
Right femoral diaphyseal fracture and radiographic follow-up. (A) At the time of fracture (December 2021). (B) Confirmed atrophic nonunion (March 2023, approximately 15 mo after the initial fracture). (C) Approximately 5 mo after revision surgery (May 2024), showing healing with callus formation.

At presentation, she had moderate sideropenic anemia (Hb 96 g/L), but she was reluctant to take iron supplements. After additional counseling and discussion of the role of iron in ADHR, the patient agreed to start ferrous(II) sulfate 80 mg BID at month 6 of burosumab treatment to correct iron deficiency and anemia and to optimize her condition ahead of the planned elective surgery. At 8 mo of treatment, the burosumab dose was increased to 50 mg (1.25 mg/kg) due to persistent hypophosphatemia, with close monitoring. The combination of the increased burosumab dose and concurrent oral iron supplementation resulted in transient hyperphosphatemia (1.61 mmol/L). Consequently, the subsequent burosumab administration was postponed by 14 d and, after 3 doses at the escalated dose of 1.25 mg/kg, the dose was reduced back to 40 mg (1 mg/kg). Treatment then continued as scheduled. Serum phosphate returned to normal at the next administration but began to decline over the next two injections, accompanied by a decrease in hemoglobin. We increased the dose of ferrous(II) sulfate to 80 mg TID. Due to menorrhagia, we recommended hormonal contraceptive therapy, which she refused. After approximately 11 mo of treatment, BMD increased further compared with the month 5 measurement by 46.6% at the LS and 36.2% at the hip. Overall, this corresponded to increases of 63.8% and 55.5% from baseline, respectively, shown in Figure 3. Trabecular bone score once again showed no substantial change.

**Figure 3:**
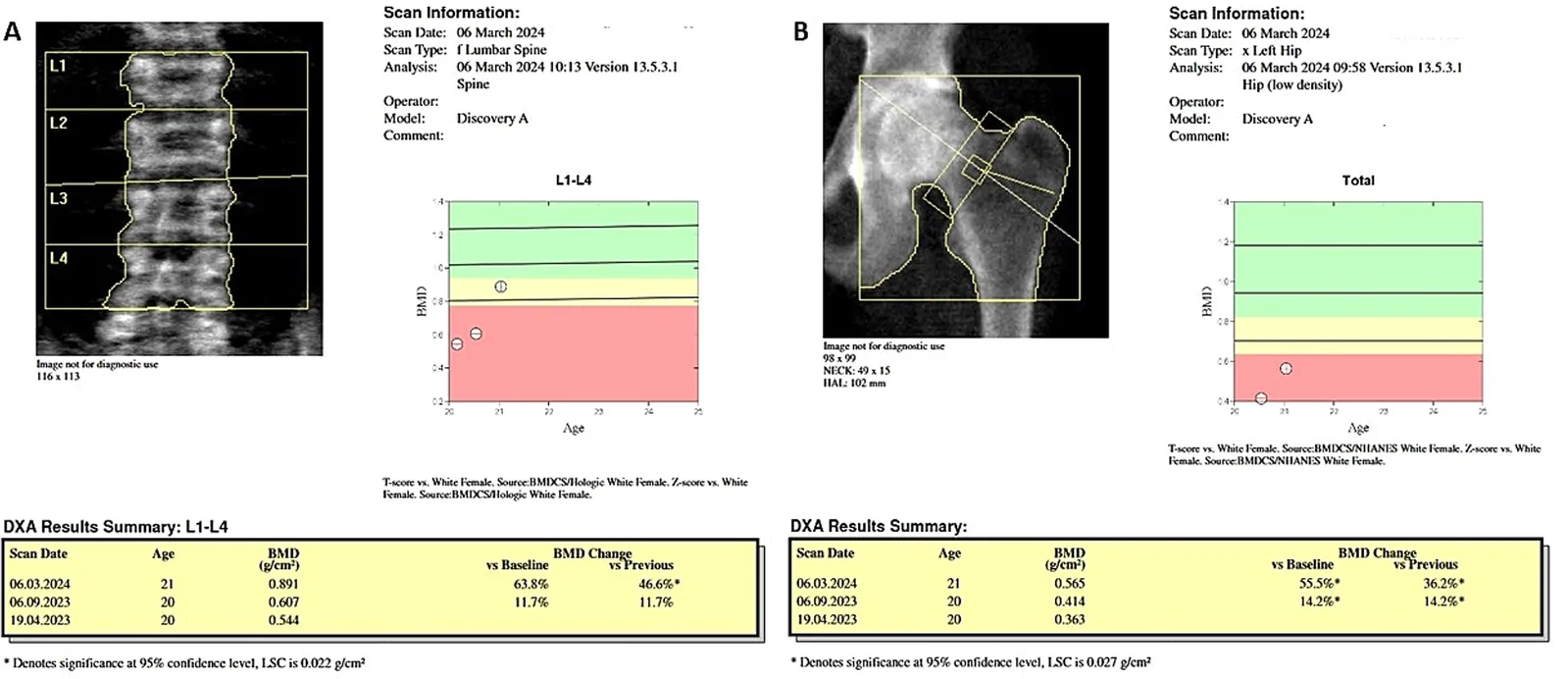
Serial DXA measurements during follow-up. (A) Lumbar spine (L1-L4). (B) Total hip. The figure shows the serial DXA reports and the corresponding changes in BMD compared with baseline and the previous measurement (dates as shown in the DXA summaries). Z-scores for this premenopausal patient are reported in Table 1, in accordance with ISCD recommendations.

## Discussion

To our knowledge, this is the first full-length report describing burosumab use in genetically confirmed ADHR with longitudinal biochemical, radiographic, and densitometric follow-up. Preliminary findings from this patient were previously presented in abstract form.^11^ The patient had severely symptomatic ADHR and sustained a fragility fracture of the femur, complicated by nonunion. Following treatment, she experienced marked symptom improvement and fracture healing progressed. Muscle strength and BMD improved, enabling the patient to regain independent mobility. While burosumab treatment was generally well tolerated, concurrent administration of ferrous(II) sulfate resulted in transient hyperphosphatemia.

To date, no prospective studies have systematically examined burosumab treatment in patients with ADHR and published evidence is limited. In our case, burosumab led to a rapid reduction in symptoms and a marked improvement in muscle strength and mobility. In patients with XLH, burosumab has been shown to enhance physical function, as measured by the Western Ontario and McMaster Osteoarthritis Index (WOMAC), and reduce stiffness, as assessed by the Short Form-36 version 2 Health Survey (SF-36v2).^12^ However, in our patient, we did not objectively measure symptom improvement using standardized instruments.

Our patient underwent additional surgical intervention, including osteotomy, osteosynthesis, and correction of the right knee contracture, which may have contributed to the overall outcome. In XLH patients, burosumab treatment has been shown to significantly improve fracture or pseudofracture healing, with a 17-fold greater likelihood of healing compared to placebo. After 24 wk of treatment, healing was observed in 43.1% of patients receiving burosumab vs 7.7% in the placebo group.^13^ Furthermore, after 48 wk, healing rates were higher in patients treated continuously with burosumab compared to those who switched from placebo (63% vs 35%).^14^

In our patient, very low BMD together with normal TBS was consistent with severe osteomalacia. In osteomalacia, bone is hypomineralized, but the microarchitecture remains largely intact.^15^ Trabecular bone score is a tool for the assessment of bone microarchitecture. It is a textural index that analyses pixel gray-level variations in LS DXA images, providing an indirect measure of trabecular microarchitecture, through the projection of the 3D structure on a 2D plane.^16^ During treatment, the patient’s BMD increased at both 5 and 11 mo, while TBS did not change. This pattern is consistent with successful bone mineralization and preserved bone microarchitecture. Analysis of transiliac bone biopsies from XLH patients revealed low and heterogeneous mineralization of cancellous bone. Treatment with burosumab for 48 wk resulted in a 35.9% increase in bone mineralization and a reduction in mineralization heterogeneity.^17^

In our patient, treatment with the standard starting dose of burosumab did not result in normalization of serum phosphate, TmP/GFR, or TRP. This differs from findings in adult XLH patients, where a dose escalation study showed normalization of these laboratory parameters in 88.5% of participants, even at doses as low as 0.6 mg/kg.^18^ Therefore, if serum phosphate does not normalize with the initial burosumab dose, a gradual dose escalation may be necessary to achieve therapeutic targets, as demonstrated in clinical trials for XLH.^18^ Consistent with this, in April 2026 the FDA updated the adult XLH prescribing information to permit dose and frequency intensification, up to 1 mg/kg every 2 wk, when serum phosphorus remains below the normal range. This corresponds to a higher cumulative exposure than the 1.25 mg/kg every 4 wk used in our patient.^19^

Importantly, in contrast to XLH, iron replacement is a key component of the management of ADHR, as iron deficiency increases FGF23 expression and worsens phosphate wasting. Addressing iron deficiency in ADHR can help to restore phosphate balance and may also influence the response to burosumab therapy.^6–8^ A more prudent, stepwise approach in iron-deficient ADHR patients would be to initiate treatment with iron replacement first. Oral iron supplementation is preferred in ADHR, as intravenous preparations, especially ferric carboxymaltose, have been associated with transient increases in FGF23 and worsening hypophosphatemia.^20^ Eventually, after repletion of iron stores, which may require a prolonged period of time, burosumab may be introduced, preferably at lower doses or with extended dosing intervals, to promote more rapid mineralization and bone healing. However, in our severely symptomatic patient, iron-first management was not feasible initially, because she was reluctant to start oral iron and rapid clinical improvement was needed. Therefore, burosumab was initiated first as bridging therapy, with iron replacement introduced once she agreed. The principal clinical benefits, namely reduced musculoskeletal pain, restored ambulation, and the first marked increase in BMD at month 5, occurred during this initial period of burosumab monotherapy, while hemoglobin remained low (87 g/L) and before effective iron repletion, supporting their attribution primarily to burosumab. In our case, the burosumab dose was increased to 50 mg with concomitant iron replacement, which resulted in TmP/GFR slightly above the reference range and caused transient hyperphosphatemia. When iron deficiency worsened in the setting of menorrhagia, and after the burosumab dose was reduced, serum phosphate and TmP/GFR declined again. Ferritin was not available at baseline. Following counseling and initiation of oral iron, hemoglobin improved from 87 to 145 g/L, supporting treatment effect. However, it later declined to 115 g/L and ferritin stayed very low (5 μg/L), most probably due to ongoing and possibly worsening iron losses from menorrhagia. Although we increased the dose of oral ferrous(II) sulfate and recommended hormonal therapy to reduce menstrual blood loss, the patient declined hormonal contraception.

Increases in bone formation markers ALP, BALP, and P1NP, and a short-term increase of CTX were observed, consistent with findings from studies in adults with XLH.^9^^,^^18^ Despite suboptimal laboratory parameters, clinical outcomes—including fracture healing, improved bone mineralization, (reflected by rising BMD and a subsequent decline in ALP/BALP), and symptom relief—showed marked improvement.

Assessing patients with ADHR has its limitations. Enthesopathies and spinal calcifications can lead to falsely elevated BMD readings, contributing to measurement uncertainty. However, a low baseline BMD, along with improvement during treatment, aligns more consistently with severe osteomalacia and enhanced mineralization. FGF23 was not measured, as the assay is not routinely available for clinical use at our institution. Nevertheless, the FGF23-mediated mechanism was confirmed genetically, while TmP/GFR and TRP provided indirect functional evidence of FGF23-mediated renal phosphate wasting. The absence of a baseline FGF23 value, together with the sequential but ultimately overlapping use of iron and burosumab, limits precise attribution of the overall phosphate and bone response to either intervention alone. In addition, burosumab is not approved for ADHR, and the dose used exceeded the adult XLH label in force at the time, so our findings reflect off-label use in a single patient and require confirmation in larger series.

## Conclusions

Because ADHR is rare, evidence for optimal treatment is limited. Iron deficiency should be corrected and ferritin monitored to guide therapy and avoid iron overload. In selected severe cases, short-term burosumab may be a useful bridging therapy to improve symptoms, bone mineralization, and fracture healing. This case adds detailed longitudinal real-world data on the use of burosumab in ADHR.

## Data Availability

The data underlying this article will be shared upon reasonable request to the corresponding author.
